# Field Application of NIR Spectroscopy for the Discrimination of the *Biomphalaria* Species That Are Intermediate Hosts of *Schistosoma mansoni* in Brazil

**DOI:** 10.3389/fpubh.2021.636206

**Published:** 2021-03-12

**Authors:** Vanessa Valladares, Célio Pasquini, Silvana C. Thiengo, Monica A. Fernandez, Clélia C. Mello-Silva

**Affiliations:** ^1^Environmental Health Monitoring and Prevention Laboratory, Instituto Oswaldo Cruz-Fiocruz, Rio de Janeiro, Brazil; ^2^Chemistry Institute, Universidade Estadual de Campinas/UNICAMP, Campinas, Brazil; ^3^Malacology Laboratory, Instituto Oswaldo Cruz-Fiocruz, Rio de Janeiro, Brazil

**Keywords:** near infrared spectroscopy, innovative technique, mollusk, freshwater snails, chemical phenotype, schistosomiasis

## Abstract

Near Infrared Spectroscopy (NIRS) is a spectroscopic technique that evaluates the vibrational energy levels of the chemical bonds of molecules within a wavelength range of 750–2,500 nm. This simple method acquires spectra that provide qualitative and quantitative data on the chemical components of the biomass of living organisms through the interaction between the electromagnetic waves and the sample. NIRS is an innovative, rapid, and non-destructive technique that can contribute to the differentiation of species based on their chemical phenotypes. Chemical profiles were obtained by NIRS from three snail species (*Biomphalaria glabrata, Biomphalaria straminea*, and *Biomphalaria tenagophila*) that are intermediate hosts of *Schistosoma mansoni* in Brazil. The correct identification of these species is important from an epidemiological viewpoint, given that each species has distinct biological and physiological characteristics. The present study aimed to develop a chemometric model for the interspecific and intra-specific classification of the three species, focusing on laboratory and field populations. The data were obtained from 271 live animals, including 150 snails recently collected from the field, with the remainder being raised in the laboratory. Populations were sampled at three localities in the Brazilian state of Rio de Janeiro, in the municipalities of Sumidouro (*B. glabrata*) and Paracambi (*B. straminea*), and the borough of Jacarepaguá in the Rio de Janeiro city (*B. tenagophila*). The chemometric analysis was run in the Unscrambler® software. The intra-specific classification of the field and laboratory populations obtained accuracy rates of 72.5% (*B. tenagophila*), 77.5% (*B. straminea*), and 85.0% (*B. glabrata*). The interspecific differentiation had a hit rate of 75% for the field populations and 80% for the laboratory populations. The results indicate chemical and metabolic differences between populations of the same species from the field and the laboratory. The chemical phenotype, which is closely related to the metabolic profile of the snails, varied between environments. Overall, the NIRS technique proved to be a potentially valuable tool for medical malacology, enabling the systematic discrimination of the *Biomphalaria* snails that are the intermediate hosts of *S. mansoni* in Brazil.

## Introduction

The planorbid snails *Biomphalaria glabrata, Biomphalaria tenagophila*, and *Biomphalaria straminea* are intermediate hosts of *Schistosoma mansoni* in Brazil. However, the identification of these species in the field is currently impossible without the dissection of specimens for the examination of the diagnostic morphological traits of the three species. The correct diagnosis of the predominant *Biomphalaria* species found in a given area is extremely important for the adoption of adequate measures for the prevention of schistosomiasis, given the epidemiology of transmission of the parasite. *B. glabrata* is considered to be the principal threat to public health of the three *Schistosoma mansoni* transmitting species found in Brazil (1), given that it is the most susceptible of the three species. In addition, that, once infected, this snail eliminates cercariae of the parasite throughout the rest of its lifetime. The three *Biomphalaria* species can usually only be distinguished by specialists based on diagnostic morphological traits, although in some cases, even these criteria may be inadequate, with reliable species identification required genetic data, using destructive procedures. Given this, more modern techniques, such as spectroscopy, may provide an important complementary approach that contributes to the identification of the species without damaging the specimen (2).

Near Infrared Spectroscopy (NIRS) is considered a simple procedure for the acquisition of spectra and both qualitative and quantitative data on the chemical components of the biomass of living organisms through the interaction between the sample and electromagnetic waves within a wavelength range of 750–2,500 nm (3–5). This treatment does not require any prior processing of the samples, does not produce dangerous residues, and it is “ecofriendly.” The species are differentiated based on the chemical phenotype of the sample. Each species has a characteristic metabolic profile determined by its role in the ecosystem, which can be detected by NIRS. Mello-Silva et al. (6) applied NIRS to the analysis of these mollusk vectors, determining the potential for the identification of the species based on the shells of specimens maintained in a controlled laboratory environment.

As the correct identification of the snails intermediate hosts of *S. mansoni* in Brazil is essential for the application of effective measures for the control and prevention of schistosomiasis, the present study evaluated a chemometric model for the inter- and intraspecific classification of these mollusks based on the comparison of spectrophotometric data on populations of the three species, both collected in the field and raised in the laboratory.

## Materials and Methods

### Snail Strain

A total of 271 live mollusks were collected from three populations in the Brazilian state of Rio de Janeiro. The *B. glabrata* specimens were collected in the municipality of Sumidouro and the *B. straminea* specimens in the municipality of Paracambi, while *B. tenagophila* was collected in Jacarepaguá, a borough of the state capital, Rio de Janeiro. These populations were selected based on Thiengo et al. (7, 8) survey of the distribution of freshwater mollusks in the state of Rio de Janeiro.

In the laboratory, 150 of the specimens collected in the field were raised in the laboratory, using water obtained from the collecting sites in which the snails were found. These individuals were exposed to a light source once a week over 30 days to verify possible natural infection by *S. mansoni*, as described in Brasil (9). None of the mollusks elected for the study were considered to be positive (infected), given that they did not eliminate any kind of cercariae. Mollusks from the same populations were maintained in aquaria in the mollusk vivarium of the Brazilian National Reference Schistosomiasis Laboratory (LRNEM) at FIOCRUZ in Rio de Janeiro, Brazil. These specimens were separated by species and population, under the same temperature conditions and provisioned *ad libitum* with fresh washed lettuce. The laboratory analyses were conducted on specimens collected recently in the field with the local water and from the snails collected from the same populations that had been raised in the laboratory. All the specimens in this study were analyzed after sexual maturity and with a shell diameter of 8–12 mm, varying according to the species.

### Near Infrared Spectrophotometry

An ABB Boomem MB 3600 near-infrared spectrophotometer with the Fourier transformation (FT-NIR) was used to collect the spectra of the live specimens of the three *Biomphalaria* species from the field and the laboratory. The mollusks were separated by species and dried externally using paper towels. The specimens were analyzed individually and randomly in transparent glass vial of 20 mL from Thermo Fisher Scientific. Each specimen was positioned at the bottom of the vial with the left side of the shell facing down and placed at the window of the apparatus for the collection of 50 spectra from each live animal, with a resolution of 16 cm^−1^. A glass vial, of the same specifications, contained spectralon was used as the spectroscopic reference for all the analyses, with the apparatus being calibrated prior to each group of samples. All the mollusks were still alive after the collection of the spectra and, in a complementary way, 20% of the animals of each group were dissected to confirm the species.

### Chemometric Analyses

The chemometric analyses were run in the Unscrambler® software, in which the raw spectra were pre-processed to reduce possible noise that may have influenced the final results. It was also necessary to create a specific spectral model for each set of samples prior to their validation. A exploratory Principal Components Analysis (PCA) was used to verify the existence of distinct clusters of samples that corresponded to the different species. Following this procedure, the results were classified using a Linear Discriminant Analysis (LDA), which is a tool that uses information from the categories associated with each standard to extract the most discriminating characteristics linearly, allowing the samples to belong to a single class (10). The table of classification produced by the LDA was used to classify the percentage of hits, that is, the number of correct classifications divided by the number of samples used for the validation (multiplied by 100 to give a percentage value).

## Results

### Intraspecific Classification

**Figure 1:**
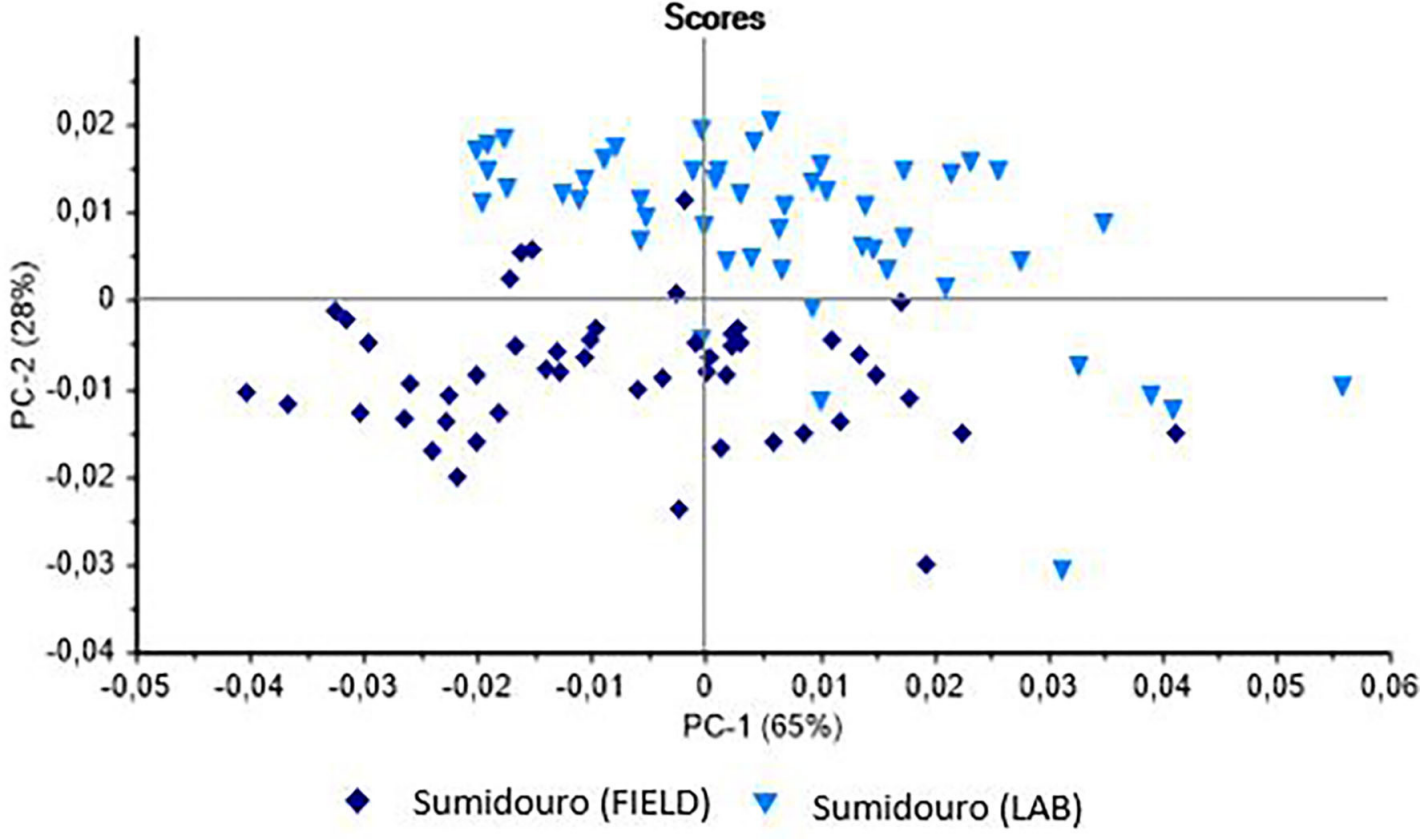
Principal Components Analysis (PCA) of the live specimens of *Biomphalaria glabrata* collected in the field in the municipality of Sumidouro (Rio de Janeiro state, Brazil) and raised in the laboratory.

Three models of classification were compiled based on the intraspecific relationships found in *B. glabrata, B. tenagophila*, and *B. straminea*, derived from the live specimens of the same populations collected in the field and raised in the laboratory. The Principal Components Analysis (PCA) of the *B. glabrata* samples (field and laboratory) is shown in Figure 1. The plot shows the clear separation of the two samples on the PC-2 axis.

**Figure 2:**
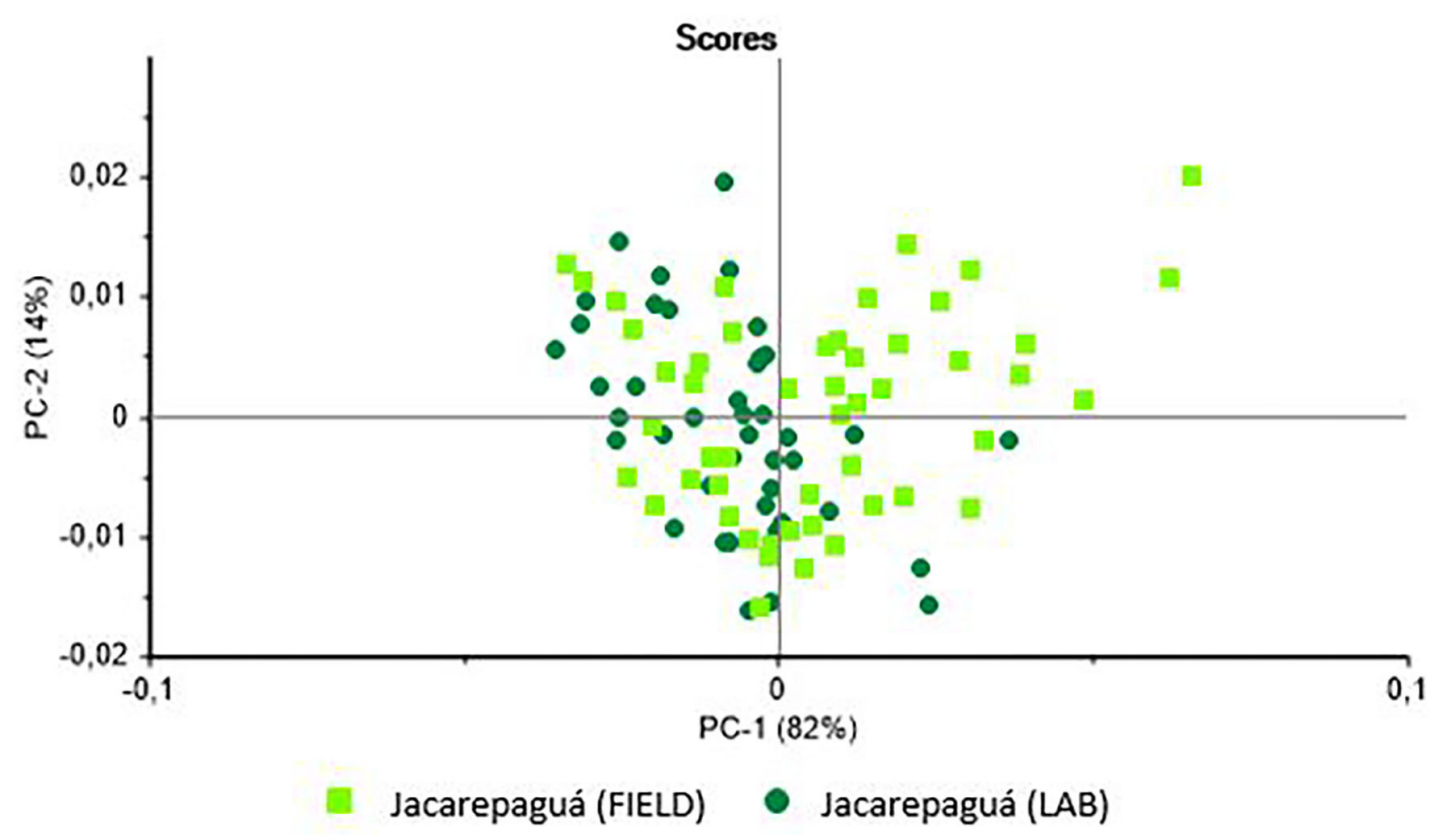
Principal Components Analysis (PCA) of the live specimens of *Biomphalaria tenagophila*collected in the field in the borough of Jacarepaguá in the city of Rio de Janeiro (Brazil) and raised in the laboratory.

**Figure 3:**
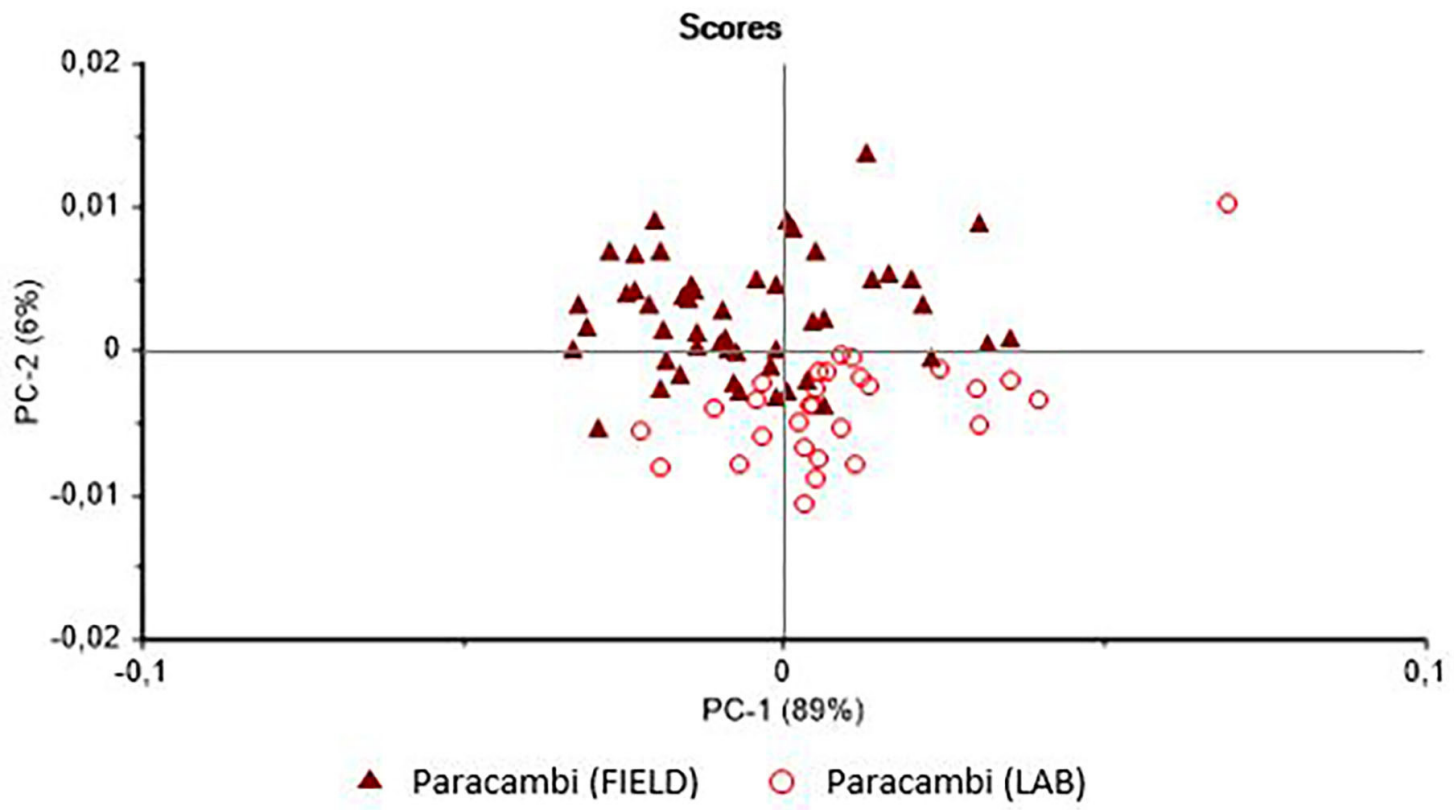
Principal Components Analysis (PCA) of the live specimens of *Biomphalaria straminea* collected in the field in the municipality of Paracambi (Rio de Janeiro state, Brazil) and raised in the laboratory.

In the case of the *B. tenagophila* population from Jacarepaguá, the analysis (Figure 2) indicated the possible separation of the two groups (field vs. laboratory). In this case, the scores separate the groups on the PC-1 axis. The PCA of the *B. straminea* samples (Figure 3) separated the two samples (field vs. laboratory) clearly on the PC-2 axis. The external validation of these classification models revealed different hit rates for each species (Table 1).

**Table 1:** Classification of the hits of the *Biomphalaria* species, based on a Linear Discriminant Analysis (LDA).

**Intraspecific analysisLaboratory vs. Field**	***Biomphalaria glabrata***	***Biomphalaria tenagophila***	***Biomphalaria straminea***
Model (*n*)	60	52	47
Validation (*n*)	40	40	32
Hits (%)	85.0	72.5	77.5

### Interspecific Classification

**Figure 4:**
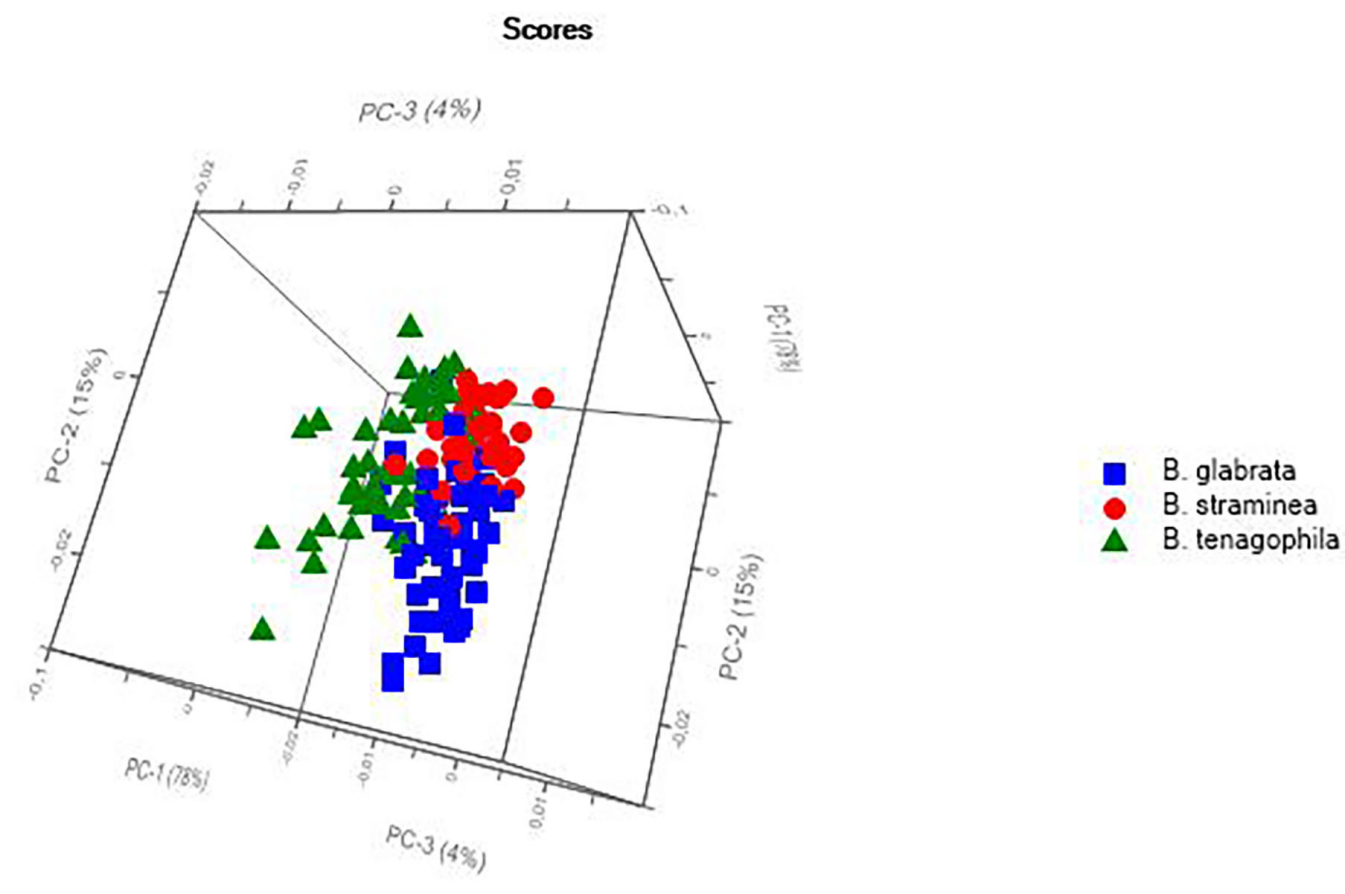
Principal Components Analysis (PCA) of the live specimens of *Biomphalaria* spp. collected in the field in Rio de Janeiro state, Brazil.

Two models were used to classify the interspecific relationships of the three *Biomphalaria* species that act as intermediate hosts of *S. mansoni*, based on the analysis of live specimens collected in the field and raised in the laboratory. The PCA of the animals collected in the field (Figure 4) presented a clear tendency for the separation of the species, albeit with some mixed scores. The external validation of the spectra of this group had a 75% hit rate, with most errors being recorded between *B. glabrata* and *B. tenagophila*.

**Figure 5:**
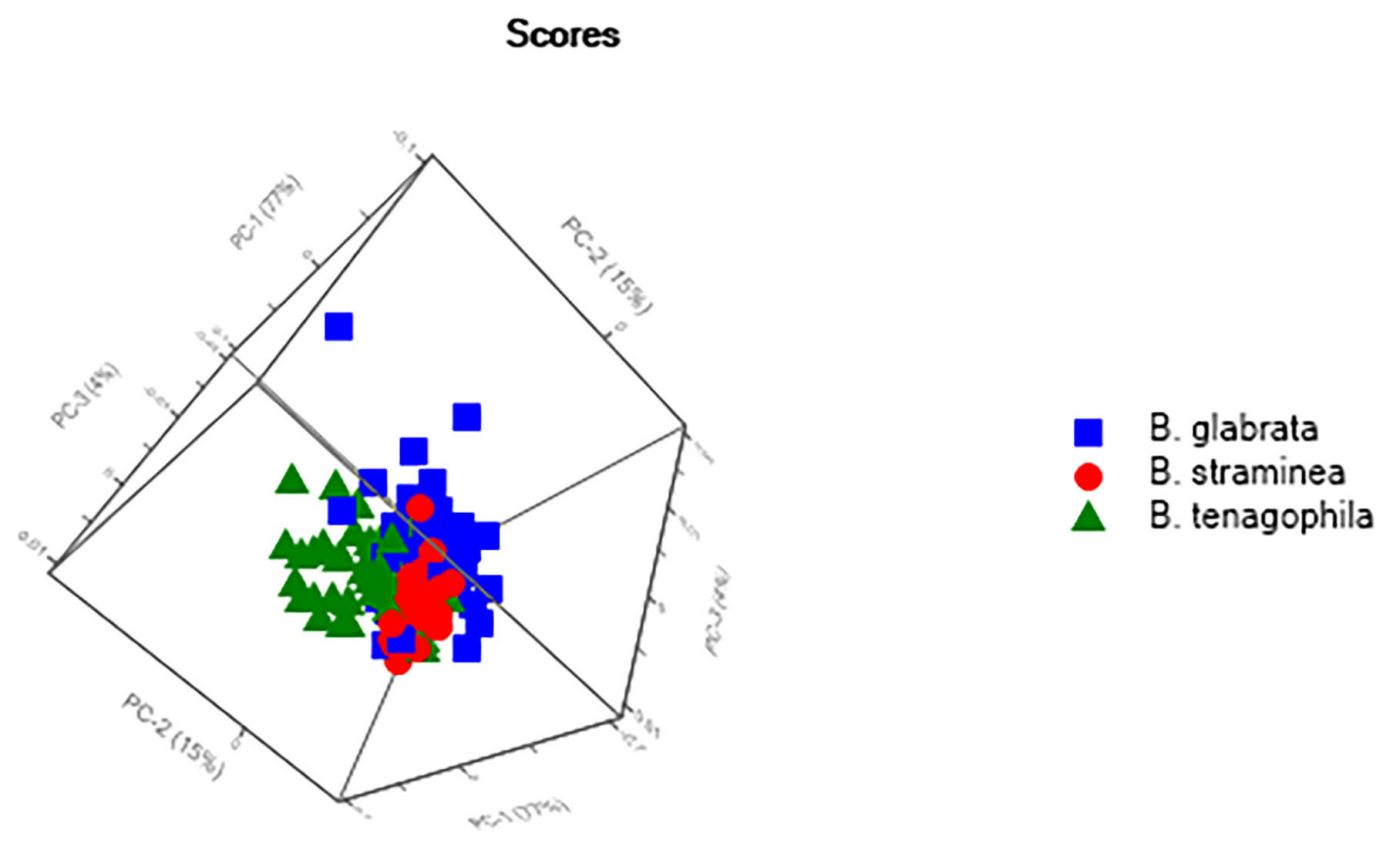
Principal Components Analysis (PCA) of the live specimens of ram's horn snail (*Biomphalaria* spp.) raised in the laboratory.

The PCA of the mollusk population raised in the laboratory (Figure 5) also revealed a tendency for the separation of species, with a hit rate of 80% for the classification of the species, as confirmed by the external validation. An interspecific analysis that included all the spectra recorded for the field and laboratory animals was also conducted to double-check the results, although in this case, there was no clear separation of the species.

## Discussion

The results of the present study indicate that the vibrational NIR spectroscopy was able to discriminate intra- and interspecific differences among the *Biomphalaria* species that are intermediate hosts of *S. mansoni*, based on the metabolic profiles presented by these snails in response to their immediate environment. The NIRS procedure provides differentiated chemical phenotypes of the specimens of the same species and different species, related to the metabolic configuration associated with the different environments, that is, the field and laboratory. As far as is known, this is the first study that has applied this approach to mollusk vectors, although Sikulu et al. (11, 12), Sikulu-Lord et al. (13), and Fernandes et al. (14) used NIRS to investigate the taxonomic, ecological, biochemical, and parasitological characteristics of mosquito vectors. Together with these analyses, the findings of the present study should contribute to the establishment of an area of research based on the application of the chemical or spectrophotometric diagnosis of the taxonomy of parasitic disease vectors. This approach should provide a valuable tool for the integrated analysis of taxonomic relationships, which complements classic taxonomic and molecular approaches.

The results of the intraspecific analyses applied in the present study emphasize the influence of the environment on the metabolism on aquatic organisms. A range of factors may have influenced the differentiation of the specimens of the same species collected in the field or raised in the laboratory.

In the wild, the snails are exposed to a wide range of contaminants and environmental impacts, which results in constant and unavoidable contact with the chemical elements dissolved in the water. *Biomphalaria* species are considered to be good indicators of environmental contamination, and have been analyzed to determine the bioaccumulation of heavy metals and metabolic shifts provoked by contact with herbicides and molluscicides (15–17). In particular, the herbicide oxyfluorfen can damage the reproductive and antioxidant systems of *Biomphalaria* species, as well as having cytotoxic and genotoxic effects (18, 19). The results of the present study demonstrated a more pronounced intraspecific pattern in *B. glabrata* (85%), when individuals from the same population collected in the field and raised in the laboratory were compared. This indicates clear differences in the metabolism of the two groups, which are possibly related to environmental factors. Mitta et al. (20) found that environmental factors had a direct impact on the relationship between *Biomphalaria* and *Schistosoma*, creating a mosaic of possible interactions. In the present study, although *B. glabrata* specimens were collected in Sumidouro, an endemic region for schistosomiasis in the state of Rio de Janeiro, none of the animals were found infected, as well as specimens of *B. tenagophila* and *B. straminea*. Pesticides are widely used on the farms in this municipality, where agriculture is a mainstay of the local economy (21), and the intoxication of local farm laborers has been reported by Oswaldo Cruz Foundation (22). Despite the evidence, complementary techniques to the NIRS, such as metabolomics and chemical analysis, are necessary to deepen the analysis in relation to environmental contamination.

The results of the present study also demonstrated the potential of NIRS for the separation and diagnosis of the three snail species without the need for the dissection of the specimens, in animals obtained from both the field and the laboratory. The identification errors found between *B. glabrata* and *B. tenagophila* indicate that the chemical phenotypes of the two species are the most similar. This may be related to the morphological and biological similarities of these two species, and their possible potential for hybridization (9, 23). However, Vidigal et al. (24) found that *B. glabrata* and *B. tenagophila* belonged to distinct clades in a phylogenetic analysis of the ITS2 molecular marker, which indicates that there is a considerable genetic distance between the two species.

Experimental metabolic studies of the three *Biomphalaria* species infected by *S. mansoni* or *Angiostrongylus cantonensis* have shown that the three species adopt distinct metabolic strategies to avoid infection. This may have contributed to the differentiation of the NIRS profiles of the three species (16, 25).

The potential for the use of NIRS to analyze the glycogen content of different types of tissue has already been evaluated in other mollusks, such as bivalves. This study (26) focused on the quality of the mollusk diet, showing variation in the glycogen content related to the condition of the mass of the viscera in different individuals of the same species. This study applied calibration models to predict the glycogen concentrations, analyzed using standard methods.

Clearly, then, NIRS has potential for use as a complementary approach for the analysis of the differentiation of the *Biomphalaria* species that are intermediate hosts of *S. mansoni* in Brazil, providing an important tool for an integrated taxonomic analysis. The potential for the differentiation of specimens at an intraspecific level nevertheless demonstrates the need to establish separate interspecific spectrophotometric models (distinguishing specimens from the field and the laboratory, for example) that guarantee the most effective and reliable diagnosis of the *B. glabrata, B. tenagophila*, and *B. straminea* specimens by NIRS.

## Data Availability Statement

The original contributions presented in the study are included in the article/supplementary material, further inquiries can be directed to the corresponding author/s.

## Author Contributions

VV collected the snails, analyzed the spectra, conceived and developed the study, and wrote and edited the manuscript. CM-S was involved in conception and development, and the writing and editing of the manuscript. CP participated in the analysis of the spectra and edited the manuscript. ST and MF contributed to the conception of the study and edited the manuscript. All authors contributed to the article and approved the submitted version.

## Conflict of Interest

The authors declare that the research was conducted in the absence of any commercial or financial relationships that could be construed as a potential conflict of interest.
